# Two species of Southeast Asian cats in the genus *Catopuma* with diverging histories: an island endemic forest specialist and a widespread habitat generalist

**DOI:** 10.1098/rsos.160350

**Published:** 2016-10-19

**Authors:** Riddhi P. Patel, Daniel W. Förster, Andrew C. Kitchener, Mark D. Rayan, Shariff W. Mohamed, Laura Werner, Dorina Lenz, Hans Pfestorf, Stephanie Kramer-Schadt, Viktoriia Radchuk, Jörns Fickel, Andreas Wilting

**Affiliations:** 1 Department of Evolutionary Genetics, Leibniz Institute for Zoo and Wildlife Research (IZW), Alfred-Kowalke Strasse 17, 10315 Berlin, Germany; 2 Freie Universität Berlin, Kaiserswerther Strasse 16–18, 14195 Berlin, Germany; 3 Department of Natural Sciences, National Museums Scotland, Chambers Street, Edinburgh EH1 1JF, UK; 4 Institute of Geography, School of Geosciences, University of Edinburgh, Drummond Street, Edinburgh EH8 9XP, UK; 5 WWF Malaysia, 1 Jalan PJS 5/28A, Petaling Jaya Commercial Centre (PJCC), 46150 Petaling Jaya, Selangor, Malaysia; 6 Institute for Biochemistry and Biology, University of Potsdam, Karl-Liebknecht-Strasse 24–25, 14476 Potsdam, Germany

**Keywords:** Felidae, Southeast Asia, last glacial maximum, Toba volcanic eruption, hybrid capture, next generation sequencing

## Abstract

*Background.* The bay cat *Catopuma badia* is endemic to Borneo, whereas its sister species the Asian golden cat *Catopuma temminckii* is distributed from the Himalayas and southern China through Indochina, Peninsular Malaysia and Sumatra. Based on morphological data, up to five subspecies of the Asian golden cat have been recognized, but a taxonomic assessment, including molecular data and morphological characters, is still lacking. *Results.* We combined molecular data (whole mitochondrial genomes), morphological data (pelage) and species distribution projections (up to the Late Pleistocene) to infer how environmental changes may have influenced the distribution of these sister species over the past 120 000 years. The molecular analysis was based on sequenced mitogenomes of 3 bay cats and 40 Asian golden cats derived mainly from archival samples. Our molecular data suggested a time of split between the two species approximately 3.16 Ma and revealed very low nucleotide diversity within the Asian golden cat population, which supports recent expansion of the population. *Discussion.* The low nucleotide diversity suggested a population bottleneck in the Asian golden cat, possibly caused by the eruption of the Toba volcano in Northern Sumatra (approx. 74 kya), followed by a continuous population expansion in the Late Pleistocene/Early Holocene. Species distribution projections, the reconstruction of the demographic history, a genetic isolation-by-distance pattern and a gradual variation of pelage pattern support the hypothesis of a post-Toba population expansion of the Asian golden cat from south China/Indochina to Peninsular Malaysia and Sumatra. Our findings reject the current classification of five subspecies for the Asian golden cat, but instead support either a monotypic species or one comprising two subspecies: (i) the Sunda golden cat, distributed south of the Isthmus of Kra: *C. t. temminckii* and (ii) Indochinese, Indian, Himalayan and Chinese golden cats, occurring north of the Isthmus: *C. t. moormensis*.

## 1. Background

Fluctuating geological and climatic conditions during the Pliocene and Pleistocene have shaped what is now recognized as the globally important Southeast Asian biodiversity hotspot. In particular, the Sunda Shelf which comprises Peninsular Malaysia, Sumatra, Borneo, Java, Bali and other smaller islands is of great interest to evolutionary biologists [1], as alternating glacial and interglacial periods resulted in the emergence and submergence of land bridges between the larger landmasses [2–4]. The impact of these transient land bridges on the distribution of genetic variation within and among species has received growing attention in the past years. For example, little or no genetic differentiation between island populations of a given species supports a scenario in which members of this species were free to move between islands during the Last Glacial Maximum (LGM) [5,6], while significant genetic divergence between mainland and Sundaic or among Sundaic populations of different islands supports a scenario of prolonged genetic isolation due to movement restriction. The latter has been demonstrated among others for murine rodents [7], common palm civets *Paradoxurus hermaphrodites* [8], clouded leopards *Neofelis* spp. [9] and leopards *Panthera pardus* [10].

A history of vicariant evolution is also ascribed to the two sister species in the genus *Catopuma*: the bay cat *Catopuma badia* (Gray, 1874) and the Asian golden cat *Catopuma temminckii* (Vigors & Horsfield, 1827). The monotypic bay cat is endemic to the island of Borneo. Despite continuous camera-trapping efforts, this carnivore remains one of the least known cat species [11]. A characteristic morphological trait of the bay cat is its pelage. It is dense reddish (bay) or grey, with gradual transitions between these forms being common [11]. Occasionally occurring black or almost black morphs have also been reported [12,13]. The bay cat is smaller than the Asian golden cat [14] and recent molecular studies showed that the two species probably split around 3.27 Ma [15].

In contrast with the island endemic bay cat, the Asian golden cat has a wide distribution in Southeast Asia, ranging from Northeast India and Nepal to southern China, Indochina and to Peninsular Malaysia and Sumatra in the Sunda Shelf. The species is polymorphic and has usually been divided into three subspecies [16,17]: *C. t. temminckii* (Vigors & Horsfield, 1827), distributed in Sumatra, Peninsular Malaysia, Indochina, Burma to Nepal; *C. t. dominicanorum* (Sclater, 1898), restricted to Southern China; and *C. t. tristis* (Milne-Edwards, 1872) with a distribution from Tibet, Sichuan to Upper Burma. In addition, it has been proposed that Asian golden cats from Yunnan should be separated as *C. t. bainesi* (Sowerby, 1924), and those from Nepal, southern Tibet and probably northwest Yunnan and west Sichuan in China as *C. t. moormensis* (Hodgson, 1831). Although colour variations, ranging from orange to black, including the blotched ‘ocelot’ type in northern populations, and size differences between northern and southern Sundaic populations have been observed (JH Mazák and ACK 2011, unpublished data), a detailed study assessing these morphological traits has not yet been conducted on the Asian golden cat. The first molecular study of the Asian golden cat included two mitochondrial genes, four autosomal genes, one X-linked and four Y-linked genes [18]. This study revealed low molecular diversity among the analysed samples, with a moderate distinction in mtDNA between animals from north of the Isthmus of Kra and Peninsular Malaysia. Autosomal genes and sex chromosome markers showed no differentiation. Sumatran samples were not included in this study.

**Figure 1.**
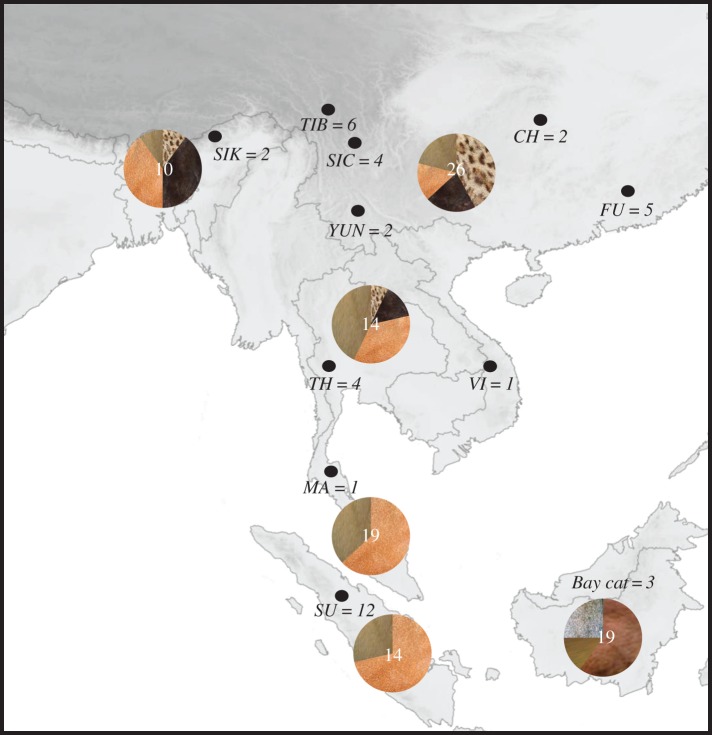
Geographical distribution of samples from Asian golden cats and bay cats used in the analyses of molecular data and pelage colour data. Pie charts represent coat colour proportions found in the population of that geographical area. White numbers on pie charts denote sample size used in pelage colour data analysis for that population. Black dots indicate populations used for mitogenome analysis. Initials of populations (*SIK*: Sikkim, India, *TIB:* Tibet, *SIC:* Sichuan, *YUN:* Yunnan, *CH:* China, *FU:* Fukien, *TH:* Thailand, *VI:* Vietnam, *MA:* Malaysia, *SU:* Sumatra) are given together with sample size.

In this study, we used in-solution hybridization capture of mainly archival samples to sequence whole mitochondrial genomes (mitogenomes). We combined these molecular data with morphological data from pelage pattern and Pleistocene species distribution projections to gain further insights into the evolutionary history of the bay cat and the Asian golden cat. Our sampling covered most parts of the respective distribution ranges (figure 1), allowing for a balanced perspective on how both species were impacted by environmental changes during the Late Pleistocene.

## 2. Material and methods

### 2.1. Samples

We obtained 38 archival samples (epithelial tissue from skulls or skins, or maxillo-turbinal bones) and two tissue samples (pathological reference sample collection of the IZW) for the Asian golden cat and we used two archival samples and one tissue sample for the bay cat (for samples and their origin see electronic supplementary material, S1).

### 2.2. DNA extraction

DNA extractions from archival samples were carried out following the Qiagen DNeasy Blood & Tissue kit protocol (Qiagen, Hilden, Germany) with an overnight lysis and a 15 min incubation period at 37°C during the elution. We included multiple extraction blanks, one per set of five samples, to control for sample cross-contamination and reagent contamination. DNA extraction and library preparation of archival samples was carried out in specially equipped laboratories dedicated to the analysis of archival samples. DNA extractions from tissue samples were carried out using the Invitek DNA extraction kit (Invitek GmbH, Berlin, Germany) in a separated laboratory dedicated to the extraction of fresh samples.

### 2.3. Library preparation and hybridization capture

Illumina libraries were prepared according to a modified paired-end sequencing protocol [19]. As the DNA extracted from archival samples was severely degraded, we used an enrichment technique (in-solution hybridization capture) to target complete mitogenomes. As none of our *Catopuma* samples was suited to generate baits for capture, we applied a cross-species capture approach [20]. Baits for cross-species capture were generated from the leopard cat (*Prionailurus bengalensis*) by amplifying three large (approx. 6 kb) overlapping regions of the mitogenome by long-range PCR (table 1); these were subsequently sheared to approximately 250 bp using a Covaris M220 (Covaris Inc., USA), purified using the QiaQuick kit (Qiagen GmbH, Hilden, Germany), and pooled equimolarly. The final steps in bait generation were blunt-end repair and ligation of biotinylated adapters (see [21], for details). In-solution capture was then carried out as described in [19]. After capture, enriched libraries were amplified using primers IS5 and IS6 [22] and purified, and then the enriched and re-amplified libraries underwent a second round of capture using freshly prepared baits because two consecutive rounds of capture significantly increased target yield (data not shown; see also [23]). Libraries were sequenced on the MiSeq platform (Illumina, San Diego, CA, USA) using MiSeq v. 3 150-cycle kits. Extraction blanks underwent the same laboratory steps (library construction and capture procedure) and were likewise sequenced to assure absence of contaminants.

**Table 1.** Primer sequences for long-range PCR designed using *Prionailurus bengalensis* NCBI reference sequence NC 016189.

primer name	sequence 5′-3′	product size
Prion_mt_F1	AAGYATTCCRCCCCAAACATAAG	6000
Prion_mt_R1	TCCTTTTTGGGTTCATTCGTAGG	6000
Prion_mt_F2	ACTAYTACTYCCCCTCCCATGA	5500
Prion_mt_R2	ATAGTGGGGCTGTTGCTTCTTC	5500
Prion_mt_F3	CAGACCTCCTAACCCTAACATGA	6000
Prion_mt_R3	TGGTAGCACGAAGATTTTTGGAT	6000

### 2.4. Bioinformatic analyses of *Catopuma* mitogenomes

Adapter sequences were clipped from paired-end reads using *cutadapt* v. 1.3 [24]. Subsequent quality trimming was performed on reads more than or equal to 20 bp using a sliding window approach and a phred quality threshold of *Q* = 20. The resulting adapter-clipped and quality-trimmed reads were merged using the software FLASH v. 1.2.8 [25]. Mapping assembly was then performed using MITObim v. 1.7 [26] using a leopard cat mitogenome sequence (GenBank accession NC 016189) as a reference for the first step of the iterative mapping. For each sample, a consensus sequence was generated in Geneious v. 8.1.2 (Biomatters, Auckland, New Zealand). Each sequence was annotated for control region, CDS, tRNAs, rRNAs and ATPase in RATT [27].

### 2.5. Phylogeographical analyses for the Asian golden cat

The dataset of 40 Asian golden cat sequences was aligned using Geneious v. 8.1.2 (Biomatters, Auckland, New Zealand). For further analysis, we excluded the control region from the alignment. PartitionFinder v. 1.1.0 [28] was applied to search for the best fitting substitution model for the dataset, with BIC (Bayesian Information Criterion) as model selector with unlinked branch length. We used HKY + I + G as substitution rate model in MrBayes v. 3.2 [29] and GTR + I + G in RAxML as it is the only model provided [30] for phylogeny reconstruction. Median-joining (MJ) haplotype networks were constructed using Network 4.6.1.3 [31]. The MJ-network is based on 40 Asian golden cat mitogenome sequences (without d-loop; 15 460 bp).

Diversity indices for the combined Asian golden cat samples were calculated in DnaSP 5.10.01 [32], including nucleotide (*π*) and haplotype (*h*) diversity, as well as transition–transversion ratios. To check for patterns of genetic variation in geographically separated populations, we divided the Asian golden cat dataset into four geographical subsets—India, Indochina, China (including Tibet) and Sumatra. These do not correspond to the currently recognized subspecies, as these are poorly defined and partly even overlap (see above), but instead correspond to zoogeographical regions of mammals in southeast Asia [33]. Two samples were excluded from this analysis: the single sample from Peninsular Malaysia, because of the geographical separation of Peninsular Malaysia and Sumatra at the Strait of Malacca since the LGM, and zoo sample CTE 2808, because its geographical origin was unknown. Pairwise *F*_ST_ values among the four Asian golden cat subsets were calculated using the algorithm implemented in ARLEQUIN 3.5 [34], significance was tested by 1000 permutations. Geographical distances between the four sample subsets were calculated using a least-cost-path (LCP) analysis applied within an LGM habitat suitability model for the Asian golden cat (see below). The LCP analysis was conducted in R (library gdistance [35]). To test if the distribution of genetic variation in Asian golden cats followed an isolation-by-distance (IBD) pattern, we applied a Mantel test implemented in R (v. 3.2.0 [36] package *ade4* [37]) and plotted genetic distances as *F*_st_/1-*F*_st_ values [38] against geographical distances using R package *ggplot2* [39].

### 2.6. Estimating divergence times

The dataset used to estimate the time to the most recent common ancestor (TMRCA) of the genus *Catopuma* included all 22 Felidae mitogenomes available in NCBI (table 2). The divergence time of the *Felidae* family (10.78 million years (Myr); CI: 8.38–14.45 Myr; [40] was set as calibration point. For tree reconstruction, we applied both a normal distribution prior with a Yule type speciation model and the HKY + I + G substitution rate model. Four independent analyses were conducted, using MCMC lengths of 100 million generations, logging every 3000th generation. All runs were evaluated in Tracer v. 1.6 for ESS > 200. LogCombiner v. 1.8.1 was then used to combine tree logs from the independent runs. The final tree log was used to resolve the phylogenetic tree, which was visualized in FigTree v. 1.4.2. (http://tree.bio.ed.ac.uk/software/figtree/). The obtained TMRCA estimate of *Prionailurus* clade (3.71 Myr (CI_95%_: 2.36–5.04 Myr)), puma lineage (4.99 Myr (CI_95%_: 3.16–6.70 Myr)), lynx lineage (3.91 Myr (CI_95%_: 2.51–5.35 Myr)) and Pantherinae subfamily (5.54 Myr (CI_95%_: 3.59–7.57 Myr)) were similar to the ones reported in a previous study [15]. Hence, we used the TMRCA estimate of *Catopuma* (3.44 Myr (CI: 2.5–4.5 Myr)) to infer divergence times at internal nodes. As the dataset included interspecies data (Asian golden cat and bay cat sequences) we applied a multi-species coalescent tree model in *BEAST with the HKY + I + G substitution rate model. Four independent runs were conducted as described above.

**Table 2.** Mitogenome sequences used for estimation of TMRCA and molecular clock rate for the genus *Catopuma*.

no.	species name	common name	NCBI accession no.
1	*Panthera pardus*	leopard	EF 551002
2	*Panthera uncia*	snow leopard	EF 551004
3	*Puma concolor*	puma	JN 999997
4	*Panthera leo persica*	Asian lion	JQ 904290
5	*Felis margarita*	sand cat	KR 132580
6	*Leopardis pardalis*	ocelot	KR 132583
7	*Lynx pardinus*	Iberian lynx	KR 132583
8	*Otocolobus manul*	Pallas's cat	KR 132585
9	*Felis catus*	domestic cat	NC 001700
10	*Acinonyx jubatus*	cheetah	NC 005212
11	*Neofelis nebulosa*	clouded leopard	NC 008450
12	*Lynx rufus*	bobcat	NC 014456
13	*Prionailurus bengalensis euptilurus*	Amur leopard cat	NC 016189
14	*Panthera onca*	jaguar	NC 022842
15	*Panthera tigris*	tiger	NC 010642
16	*Lynx lynx*	Eurasian lynx	KM 982549
17	*Prionailurus viverrinus*	fishing cat	KR 135742
18	*Prionailurus rubiginosus*	rusty-spotted cat	KR 135744
19	*Prionailurus planiceps*	flat-headed cat	KR 135743
20	*Catopuma temminckii*	Asian golden cat	KR 135745
21	*Catopuma badia*	bay cat	KR 135746
22	*Pardofelis marmorata*	marbled cat	KT 288227

### 2.7. Demographic analysis

To reconstruct the demographic history of the Asian golden cat, we used the TMRCA derived from the analysis conducted for the *Catopuma* genus (see above) with lognormal distribution as prior to determine root model height. Coalescent extended Bayesian skyline (EBSP) was selected as tree prior as this analysis concerned intraspecific variation. Analyses were conducted with MCMC lengths of 30 million generations, logging every 1000th generation in BEAST v. 1.8. Each run was evaluated for ESS > 200 in Tracer v. 1.6. Results were plotted as skyline plots using R package *ggplots2*.

### 2.8. Projection of Pleistocene Asian golden cat and bay cat distributions

We used the approach described in [10] to project the Pleistocene distribution of the Asian golden cat and the bay cat. Current distribution ranges were taken from the IUCN/SSC Red List of Threatened Species. As a study area, we used the region between 87° E and 131° E longitude and between of 34° N and 12° S latitude, to ensure that the species distribution modelling is linked to the climatic conditions in South and Southeast Asia. We randomly selected 10% of the rasterized distribution ranges for the Asian golden cat (8302 occurrences) and for the bay cat (1261 occurrences) to serve as occurrences for model-fitting procedures, and then pseudo absences (*n* = 8310 for Asian golden cat, *n* = 1270 for bay cat) were sampled from the whole remaining study area. To avoid multicollinearity, only environmental predictors with −0.7 < *r* < 0.7 (Pearson's correlation) were retained for model building, resulting in three predictors for the Asian golden cat (‘min. temperature of coldest month’, ‘temperature annual range’, ‘precipitation of wettest quarter’) and four predictors for the bay cat (‘max. temperature of warmest month’, ‘temperature annual range’, ‘precipitation of driest month’, ‘precipitation of wettest quarter’). We then built an ensemble model by taking the mean of the probabilities predicted by 18 models: three random presences sets fitted with the two algorithms (MAXENT and GBM) and with three cross-validation runs. All statistical analyses and shape file and raster manipulations were carried out using R packages *maptools* [41], *rgdal* [42] and *raster* [43].

### 2.9. Pelage coloration

In total we analysed the pelage colour variation data of 83 Asian golden cat individuals (52 museum specimens, 30 camera-trap photographs and 1 zoo sample with known origin) and 19 bay cat individuals (photographs of 8 museum specimens, 9 camera-trap photographs and 2 published accounts; [44,45]). To compare pelage variation within the Asian golden cat populations from different geographical origins, we used the geographical sample subsets created above—India, China (including Tibet), Indochina and Sumatra, and added a fifth one: Peninsular Malaysia—to estimate geographical coat colour frequencies (figure 1). We defined four coat colours—blotched, black or grey, red, and brown to divide samples into their corresponding colour or pattern (see electronic supplementary material, figure S2). The obtained frequencies were used to calculate a matrix of coat colour distances between the geographical regions. We then employed a Mantel test implemented in R to test whether the coat colour distances between the geographical regions correlated with the LCP distance (see above). Based on these distance matrices, we also calculated a cluster dendrogram using the R package *dendextend* [46].

## 3. Results

**Figure 2.**
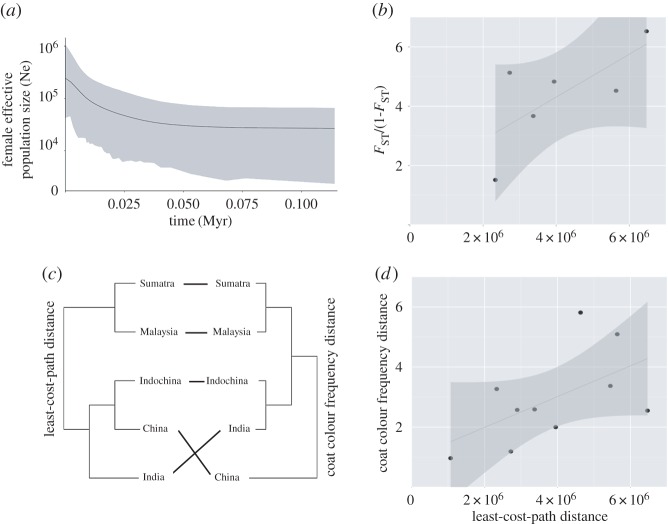
Population demographic analysis, isolation-by-distance analysis using genetic data, coat colour frequency and least-cost-path (geographical distance) data for the Asian golden cat population; (*a*) Extended Bayesian Skyline Plot for the Asian golden cat population; *x*-axis displays times in million years, *y*-axis displays effective population size Ne in log scale where Ne /*τ* (*τ* = 6.5 years × 10^6^). (*b*) Graph represents correlation between Slatkin's distance (*F*_ST_/1-*F*_ST_) and least-cost-path distance among geographical populations; (*c*) dendrogram comparison between coat colour frequency and least-cost-path distance; (*d*) graph represents correlation between coat colour and least-cost-path distance between geographical populations.

We obtained mitogenomes (16 471 bp) for 3 bay cats and 40 Asian golden cats with a minimum coverage of 5×. Each position with coverage less than 5× was replaced with *N* (approx. 1–2% of all positions per sample) before phylogenetic and phylogeographical analyses. These mitogenome sequences were translated into protein sequences to verify the alignment based on coding frames. We also compared our sequences with the complete mitogenome of the Asian golden cat stored in the NCBI database (Accession ID KP202267) to confirm their mitochondrial origin. All mitogenomes represented individual haplotypes, indicating maternal unrelatedness of all samples. In total 787 fixed mutations separated the bay cat and the Asian golden cat mitogenomes, and among the 3 bay cat and 40 Asian golden cat mitogenomes we found 33 and 224 variable positions, respectively. The MJ-haplotype network for mitogenomes (electronic supplementary material, figure S1) showed a minimum of 44 mutations between haplotypes from Indochina (haplotypes H16--H40) and Sumatra (H3--H14), a minimum of 27 mutations between Indochina and Peninsular Malaysia (H15) and a minimum of 19 mutations between haplotypes from Peninsular Malaysia and Sumatra. It is also noteworthy that the two samples from Sikkim (H1, H2) were separated from all other haplotypes by at least 41 mutations. Nucleotide diversity among Asian golden cat mitogenomes was *π* = 0.003 (s.d. = 0.0023). We found a moderate, yet not significant, correlation between genetic distances among geographical subsets and their geographical distances (*r*^2^ = 0.70731, *p* = 0.1238; figure 2*b*).

### 3.1. Phylogeography

**Figure 3.**
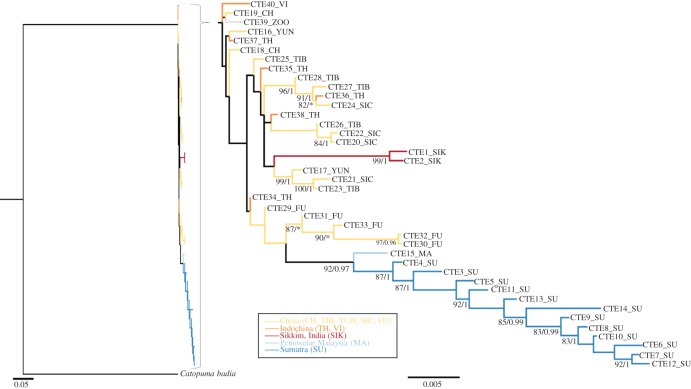
Maximum-likelihood phylogenetic tree derived from Asian golden cat mitogenomes using the bay cat (*Catopuma badia*) as outgroup. The Bayesian phylogenetic tree provided identical topologies. Support values for nodes were obtained from ML analysis (RaXML) and Bayesian inference (MrBayes). Only values greater than 80% (RaXML) and greater than 0.9 (posterior probability values for Bayesian trees) are shown. Smaller values are denoted with asterisk. Haplotypes and their origins are listed separately (electronic supplementary material, S1).

For the Asian golden cat dataset, trees obtained by ML (maximum-likelihood) and BI (Bayesian inference) were concordant in their topologies (figure 3). Sumatran haplotypes (SU; figure 3) formed a monophyletic clade. Indian (Sikkim, SIK) haplotypes and Chinese haplotypes from Fukien (FU) also formed distinct monophyletic groups. The other samples from China (Yunnan, YUN; Tibet, TIB; Sichuan, SIC) as well as those from Thailand (TH) were paraphyletic; individuals from the same provinces were found in different clades. This indicates some recent gene flow within China but also between China and Indochina as Chinese samples were not clearly distinct from Indochinese samples.

### 3.2. Molecular dating

**Figure 4.**
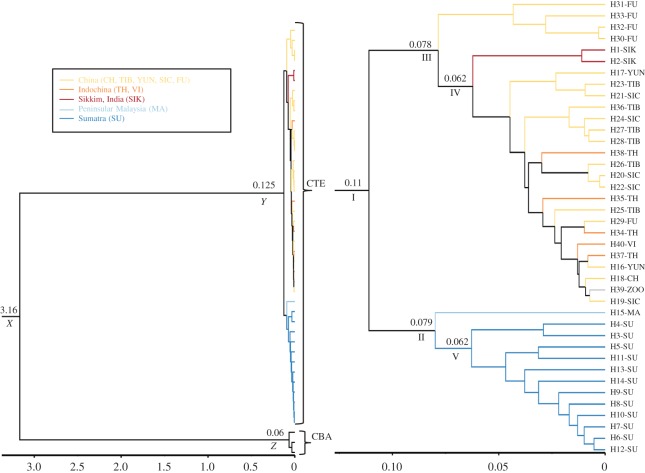
Divergence time estimates plotted onto a phylogenetic tree reconstructed using BEAST. Left: *Catopuma* genus-tree showing molecular dating for the Asian golden cat and the bay cat (CBA: *Catopuma badia*, CTE: *Catopuma temminckii*). Right: CTE species tree showing molecular datings within the Asian golden cat. Only nodes supported with posterior values = 1 are displayed with their age in million years (for the genus tree: nodes X, Y, Z; for the species tree nodes I--V). CI_95%_ values for nodes X, Y and Z and nodes I–V are given elsewhere (table 3).

As there were no *Catopuma* fossils available for age calibration, we used the divergence time of the family Felidae [40] and calculated a molecular rate of 0.0133 substitutions per site per Myr (s.d. = 0.00288) using 22 mitogenomes of felids (table 2; electronic supplementary material, figure S3). Based on this rate and the number of differences, we estimated a divergence time between Asian golden cat and bay cat of approximately 3.16 Myr (CI_95%_ = 2.05–4.54 Myr) (table 3 and figure 4). This is slightly more recent than what we had used from Felidae mitogenomes (3.44 Myr (CI: 2.5–4.5 Myr)) though it still falls in the CI_95%_ range of the splitting time reported in a previous study [15]. Based on this date, we estimated the intraspecific divergence time for the Asian golden cat clades to be approximately 112 kyr (CI_95%_ = 77–151 kyr) (figure 4). The Bayesian skyline plot revealed a very recent population expansion for the Asian golden cat (figure 2*a*).

**Table 3.** Estimated divergence date with 95% confidence interval (CI_95%)_ and posterior values as a node support (figure 4).

node	time in Myr	CI_95%_ in Myr	posterior value
dating for *Catopuma* genus			
X	3.167	2.052–4.548	1
Y	0.125	0.076–0.188	1
Z	0.066	0.0367–0.104	1
dating for the Asian golden cat internal nodes			
I	0.111	0.077–0.151	1
II	0.079	0.054–0.112	1
III	0.078	0.052–0.11	1
IV	0.062	0.041–0.087	1
V	0.062	0.04–0.08	1

### 3.3. Projection of Pleistocene Asian golden cat and bay cat distributions

**Figure 5.**
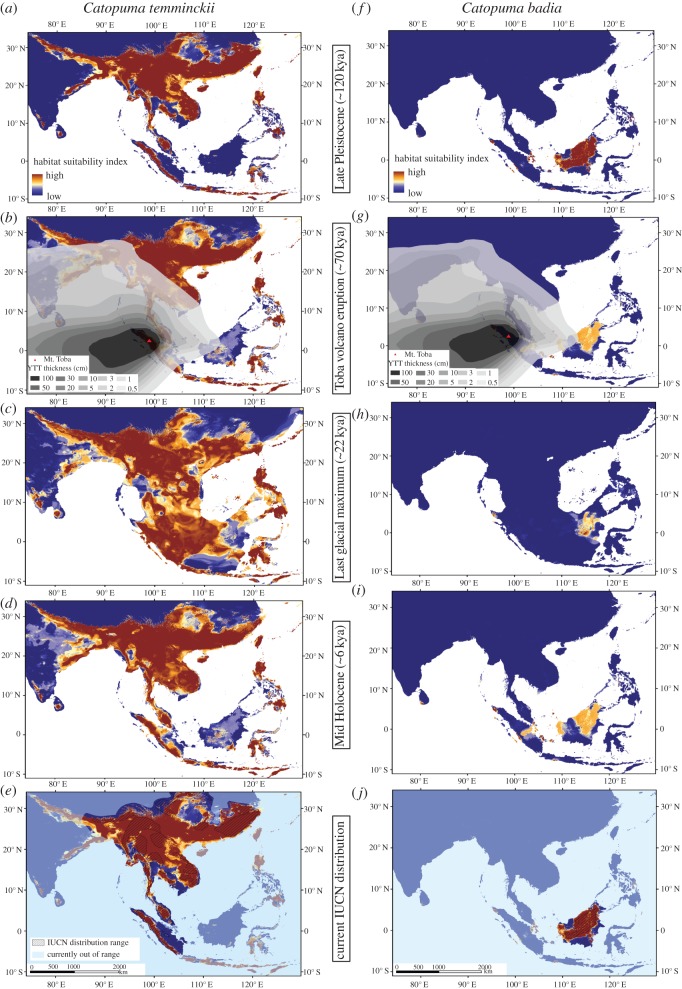
Projected distribution of the Asian golden cat (*a–e*) and the bay cat (*f–j*) along a time axis from 120 kya until present: (*a,f*) for the Late Pleistocene (approx. 120 kya) using the LGM projections; (*b,g*) the thickness of the Young Toba Tuffs (YTT) was superimposed on the projection to indicate the severity of impact of the Toba super volcanic eruption approximately 74 kya; (*c,h*) the Last Glacial Maximum (approx. 22 kya); (*d,i*) the mid Holocene (approx. 6 kya) and (*e,j*) the current distribution according to the IUCN Red List of Threatened Species used for the modelling (hatched pattern), while areas outside of the current distribution were shaded in light blue.

Habitat distribution modelling (figure 5*a–e*) indicated that large parts of Southern China contained suitable habitat for the Asian golden cat throughout the Late Pleistocene. In particular, during the LGM, most parts of the exposed Sunda Shelf were habitable, including Sumatra and Peninsular Malaysia. However, areas on Borneo, in particular in northeastern Borneo, were only marginally suitable as habitat. Although the area of suitable habitats in the Sunda Shelf receded with increasing temperatures and rising sea levels at the beginning of the Holocene, large areas in Peninsular Malaysia and Sumatra remained suitable for the Asian golden cat.

By contrast, projections for the bay cat (figure 5*f–j*) suggested that this species was probably confined to Borneo throughout the Late Pleistocene. Its range was particularly restricted during the LGM, despite the greater available Sunda land masses.

### 3.4. Pelage coloration

The bay cat displayed three different morphs—grey, red and brown (figure 1). Among Asian golden cats, the greatest diversity in coat coloration was observed in China, Tibet, Northeast India and Indochina, with the occurrence of all four recognized morphs: red, brown, spotted and black. By contrast, in Peninsular Malaysia and Sumatra only the red and the brown morphs were observed (figure 1). We found a moderate correlation between coat colour and our LCP distance matrix (*r*^2^ = 0.5604, *p*-value = 0.064; figure 2*d*). This correlation is supported by the similar topologies in the dendrograms of the two distance matrices (figure 2*c*).

## 4. Discussion

### 4.1. Evolution history of the bay cat and the Asian golden cat

The Asian golden cat and the bay cat are allopatric species of the genus *Catopuma*. Based on our mitogenome data, we estimated that Asian golden cat and the bay cat split during the late Pliocene approximately 3.16 Ma, which is somewhat more recent than had been estimated before [15]. At this time, Borneo was still connected to other parts of the Sunda Shelf and Southeast Asia. During the Late Pliocene, however, the Isthmus of Kra was submerged by high sea levels for an extended period of time [47], geographically dividing mainland Southeast Asia and Sundaland. This Late Pliocene vicariance event is considered to be one driver of speciation in Southeast Asia, because numerous other species split during this time, e.g. rodents [7], *Macaca* spp. [48,49], masked palm civet [5] and common palm civet [8]. In the case of the two *Catopuma* species, it is conceivable that following the split into the northern Indochinese ( = *temminckii*) and the southern Sundaic ( = *badia*) populations, the latter specialized and became more adapted to tropical evergreen rainforests, whereas the northern population remained a more generalistic species due to the higher habitat diversity in Indochina. Subsequently, the two emerging species responded differently to the changing environmental conditions that prevailed during the Late Pliocene and Pleistocene.

During the glacials of the Pleistocene, Sunda Shelf evergreen rainforests became restricted to smaller areas due to a cooler and drier climate, particularly during the LGM [50]. Our species distribution projections indicated that the bay cat had probably been constrained to the rainforest refugia in central and northern Borneo (figure 5*h*) because the more open savannah-like habitats of western and southern Borneo [1,3,51] were unsuitable for this specialized forest dwelling species. This rainforest refugium scenario was also proposed for other Bornean species, such as colobine monkeys [52] and termites [53]. However, due to the very small sample size (*N* = 3) and the restricted distribution coverage of the bay cat samples available for molecular analyses (all were from northeastern Borneo), we could not reconstruct the population history of the species and thus we do not have the molecular means to infer potential population size fluctuations, i.e. whether the species had experienced a population bottleneck (e.g. during the LGM) and/or underwent a subsequent population size expansion. Therefore, more extensive, ideally Borneo-wide sampling is required to test the hypothesis of an LGM refugium in the rainforests of northeastern Borneo, as suggested by our species distribution model (figure 5*f–j*).

In contrast with its sister species, the Asian golden cat could have expanded its distribution range southward to the Sunda Shelf using land bridges that were temporarily available during glacial periods of the Pleistocene (figure 5*a*). Given the intraspecific clade split at approximately 112 kya, such a southward movement to Peninsular Malaysia and Sumatra probably took place during Late Pleistocene (126–11 kya), and presence of Asian golden cats on Sumatra at that time is supported by Late Pleistocene/Early Holocene fossils from the Lida Ayer cave in West Sumatra [54]. Our molecular data on population demography favoured a late Pleistocene (approx. 30–25 kya) population expansion of the Asian golden cat on Sumatra (figure 2*a*). We cannot exclude that Asian golden cats had reached Sumatra much earlier (shortly after the clade split), but the full colonization of Sumatra, evidenced by population expansion, took place much later. This disparity can be explained by local extinction of these ‘potential early arriving’ Asian golden cats on Sumatra and Peninsular Malaysia. Such extinction may have been the result of maladaption to rapidly changing environmental conditions during the succession of glacials and interglacials in the Late Pleistocene. However, such a scenario is not supported by the current distribution of Asian golden cat, which lives in habitats with diverse environmental conditions reflecting the large adaptive potential of this species. Instead, it is conceivable that the Toba super volcanic eruption on Sumatra approximately 74 kya, which has also been linked to the local extinction of other species (orangutans *Pongo* spp. [55], clouded leopards [9], tigers [6]), also impacted Asian golden cat populations (figure 5*b*). A probable post-Toba expansion of Asian golden cats from southern China and northern Indochina, areas which were less or not affected by Toba, is also supported by the sample from Peninsular Malaysia, which is the basal-most branch of the Sundaic clade and based on the network (electronic supplementary material, figure S1) and phylogenetic tree (figure 3) genetically positioned between samples from Mainland Indochina and those from Sumatra. Such a scenario—post-Toba population expansion from south China to the Sunda Shelf, accompanied by low nucleotide diversity due to the short evolutionary time frame—has also been reported for tigers [6,56].

While samples from Sumatra formed a monophyletic cluster, samples from different provinces/regions in China and Indochina did not form respective regional clusters in our phylogenetic reconstructions (figure 3), suggesting past gene flow between these populations. By contrast, samples from Sumatra, Peninsular Malaysia, Sikkim (India) and Fukien (China)—the most southern, most western and most eastern parts of the distribution range—were separated from the other Indochinese and Chinese samples indicating spatial differentiation. The tree-like pattern of the Sumatran samples in the haplotype network (electronic supplementary material, figure S1) indicated a directional north-to-south expansion of golden cats after a single colonization. However, multiple colonization events on Sumatra from Peninsular Malaysia cannot be excluded. To test these scenarios additional samples from Peninsular Malaysia and from Sumatra with precise locality information would be needed. A haplotype radiation from a few founders in Central Sumatra would have resulted in a star-like pattern [57] and is thus rather unlikely.

The southward expansion of Asian golden cats is also supported by the pelage data. Southern Chinese populations showed the greatest diversity and an almost even proportion of all four colour morphs (figure 1). Frequencies of blotched and melanistic morphs decline towards the Sundaic population, which completely lacked these morphs and was characterized by red and brown golden cat individuals only. The pronounced morphological diversity of Asian golden cats on the mainland relative to their Sundaic conspecifics may be the result of selection over a long time and can be interpreted as local adaptation to the more diverse habitats [58]: melanistic golden cats mostly occur in temperate subalpine and alpine habitats (elevation up to 5000 m.a.s.l.) in northeast India [13], while spotted golden morphs mostly occur in dry deciduous forests, tropical savannahs, grasslands and occasionally shrublands [16]. As the southward population expansion of the Asian golden cats to the Sunda Shelf was accompanied by an increased frequency of red and brown golden morphs (the subtropical habitats became more homogeneous), the probability also increased that these high frequency morphs would be the ones colonizing the Sunda Shelf.

For the bay cat, three different coat colours (red, brown and greyish black) have been recorded intermixed from different regions of Borneo. In contrast with the Asiatic golden cat's coat colour morphs, which are highly contrasting and distinctive, the bay cat's polymorphism is tonally neutral. Close examination of the pelage of the greyish black morphs show an underlying reddish coloration (e.g. FMNH 8378) and we suspect that some individuals may change coloration during their lives as has been recorded for the polymorphic African golden cat, *Caracal aurata* [59] Given that most of the bay cat's probably mammalian predators and prey have dichromatic vision, this means that these colour variants are not visually distinct from each other, especially in the low light levels of closed-canopy forests. Therefore, this observation supports the species distribution projections, which suggested that the bay cat has probably been restricted to more homogeneous evergreen rainforests during the Pliocene and Pleistocene. By contrast, the brighter Asiatic golden cat morphs could be seen as an adaptation to open deciduous forests.

### 4.2. Taxonomy of the Asian golden cat

Several different species and subspecies of Asian golden cat have been described based on different colour morphs [17], e.g. *Felis tristis* for the ocelot-like coat pattern. Thus, we expected the phylogeny (figure 3) to show clades consisting of particular colour morphs, reflecting their putative subspecies assignments. Although there was a clear reduction of colour morphs towards Peninsular Malaysia and Sumatra, the Asian golden cats cannot be assigned to any population based on their coloration (no colour morph was specific to any particular population). Besides rejecting the colour-morph based classification, we also found an indication that modern Asian golden cats expanded only very recently. Therefore, such a recent expansion provides poor support for recognizing any subspecies of Asian golden cat and instead suggests it should be regarded as being monotypic.

On the other hand, our molecular data showed that both the Indian and the Peninsular Malaysia/Sumatran populations were distinct from all others. We could, however, not distinguish the Indian population from Indochinese or Chinese ones based on occurrence of colour morphs, so that an Indian subspecies is likewise not supported.

As already pointed out, Asian golden cats from Sumatra and their conspecifics from the mainland formed two well-separated clusters with the sample from Malaysian peninsula in between. A split between the Asian golden cats from Peninsular Malaysia and those from Indochina had already been suggested in an earlier study [18], and also the absence of certain colour morphs in Peninsular Malaysia and Sumatra supports a distinction of these Asian golden cats from their mainland relatives. Considerable body size differences (ACK 2011, unpublished data) between Sunda and Indochinese/Chinese/Indian Asian golden cats further support the distinction of two subspecies, one occurring north of the Isthmus of Kra and the other one south of it:

(1) North of Isthmus of Kra: *Catopuma temminckii moormensis*, distributed in Indochina (Thailand, Cambodia, Lao and Myanmar), China, Tibet, Nepal and northeast India (Sikkim). Four different colour morphs: blotched, black, red and golden brown are recorded throughout the distribution range.
(2) South of Isthmus of Kra: *Catopuma temminckii temminckii*, distributed in Peninsular Malaysia and Sumatra. Two dominant colour morphs: red and golden brown. So far, no blotched morphs have been reported from Sumatra/Peninsular Malaysia. Melanistic black individuals were not in our sample collection but have been seen occasionally [11].

## 5. Conclusion

Phylogenetic analyses of mitogenomes, habitat distribution models and analysis of pelage colour data of the Asian golden cat and the bay cat presented here helped to elucidate the evolutionary history of these sister species. We argue that the flooding of the Isthmus of Kra in the Pliocene and subsequent climate and vegetation variations between Sundaland and Indochina have caused a species split between the bay cat and the Asian golden cat lineages approximately 3.16 Ma. Our data suggest that the bay cat became restricted to northern Borneo during the Pleistocene and particular in the LGM, when evergreen rainforest habitats were confined to that region. As a habitat specialist for the closed evergreen rainforest, the bay cat is thus much more susceptible to extinction than its sister species, the Asian golden cat, a generalist species with a much larger distribution across different habitat types. Although its recent population expansion, its low intra-population nucleotide diversity and the cline of variation in pelage colour depict the Asian golden cat as being monotypic, we argue that a recognition of two subspecies is warranted based on the presence of two distinct mitogenomic clades, the absence of certain colour morphs in the Asian golden cats from Peninsular Malaysia and Sumatra, and the considerable size differences between Sunda and Indochinese/Chinese/Indian Asian golden cats. As a conservative approach, we recommend to collapse the 3–5 subspecies and to treat the Asian golden cat as a species with just two subspecies until further evidence (e.g. from Peninsula Malaysia) suggests otherwise.

## Supplementary Material

Additional File 1 : it contains Sample details and GenBank accession number

## Supplementary Material

Figure S1: Haplotype network

## Supplementary Material

Figure S2: Coat colour of asian golden cat

## Supplementary Material

Figure S3: Phylogenetic tree with molecular dating

## Data accessibility

Mitogenome sequences produced in this study are deposited in GenBank under the accession numbers for the Asian golden cat KX224490-KX224529 and for the bay cat KX265094-KX265096 (see electronic supplementary material, S1). Also, mitogenome alignments are submitted at Dryad under: http://dx.doi.org/10.5061/dryad.472b9 [60].
