# Supplementary figures and images for: Two species of Southeast Asian cats in the genus Catopuma with diverging histories: an island endemic forest specialist and a widespread habitat generalist
Source: R Soc Open Sci. 2016 Oct 19;3(10):160350. doi: 10.1098/rsos.160350 (PMC5098974; doi:10.1098/rsos.160350)

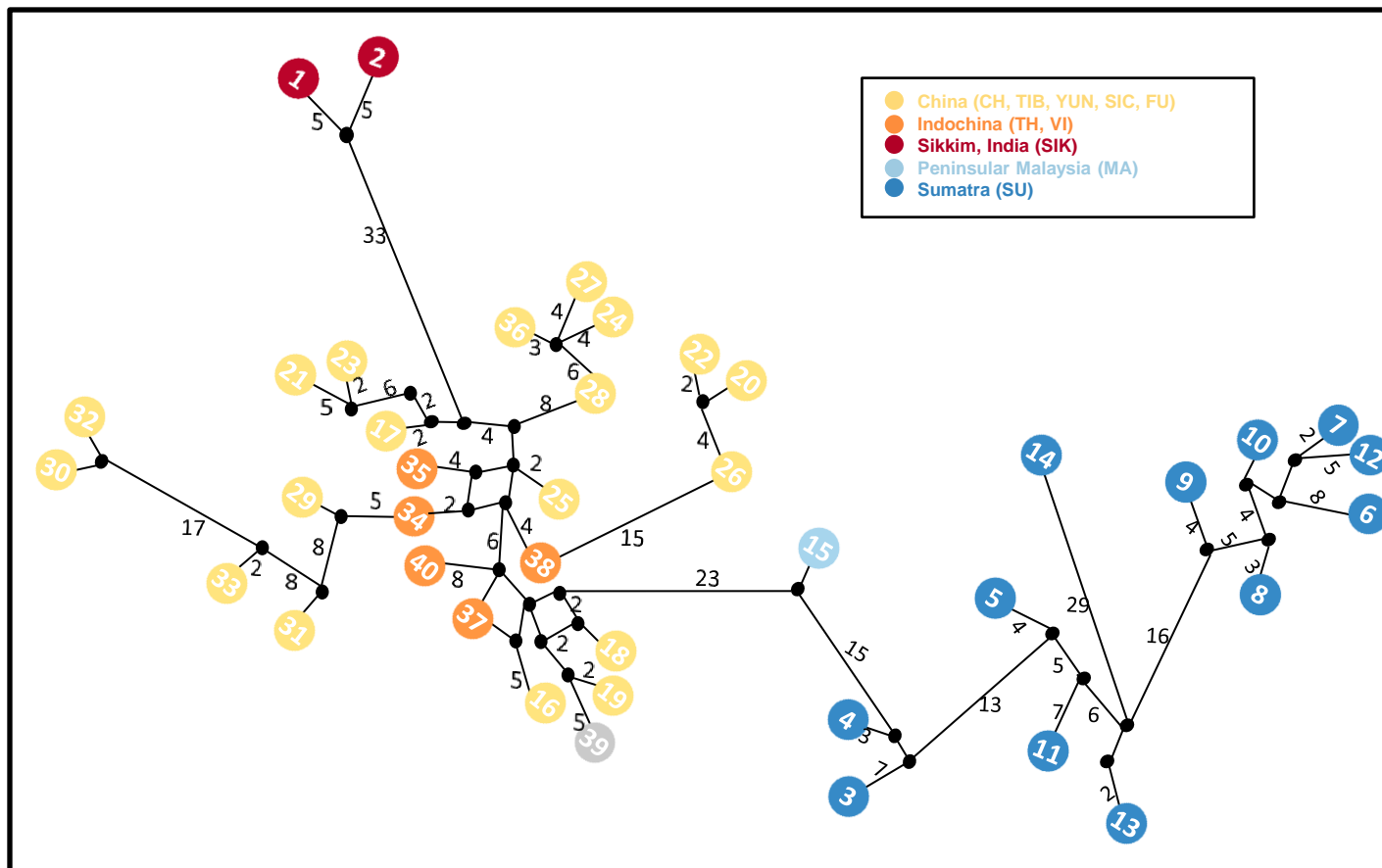

Supplement: Figure S1: Haplotype network [file rsos160350supp2.pdf]

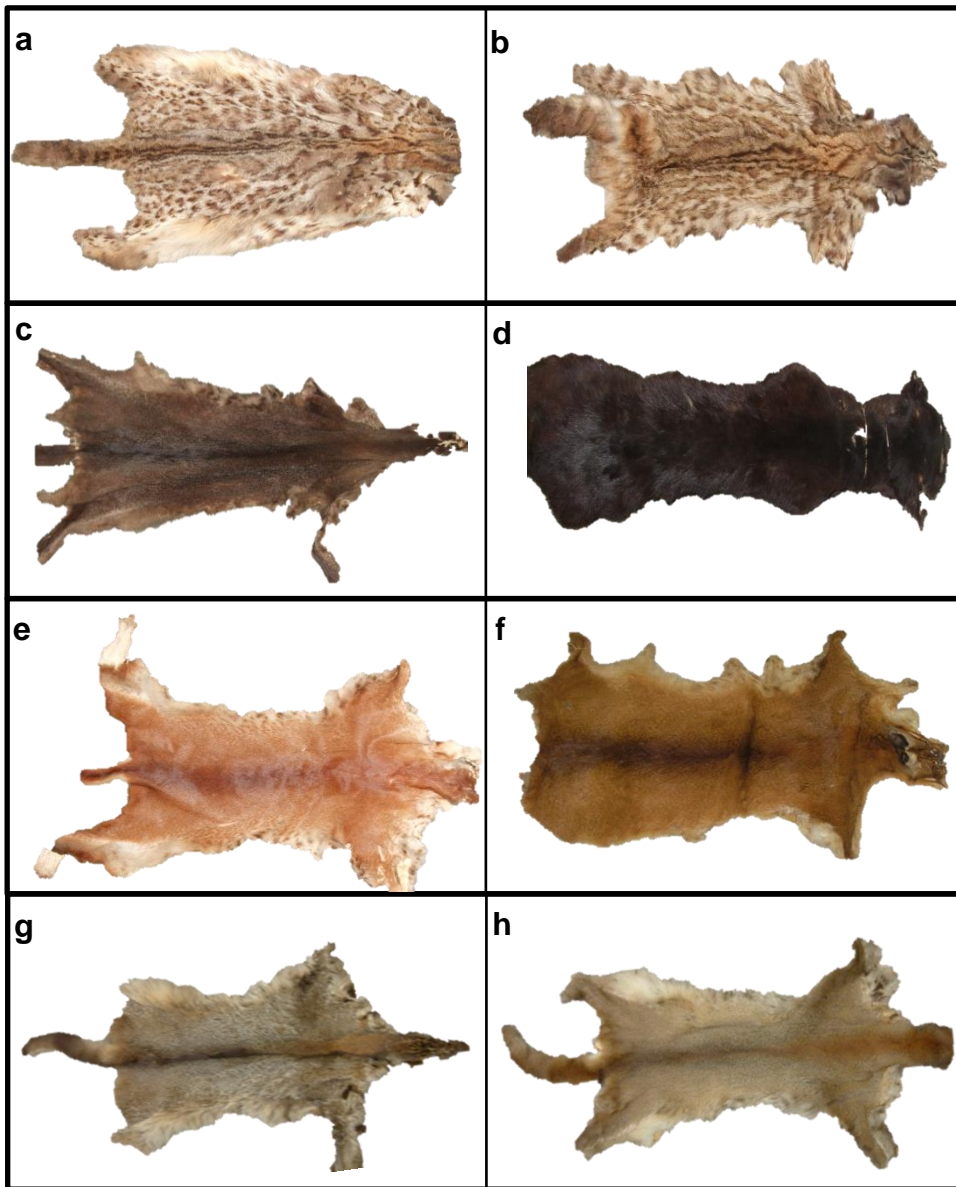

Supplement: Figure S2: Coat colour of asian golden cat [file rsos160350supp3.pdf]

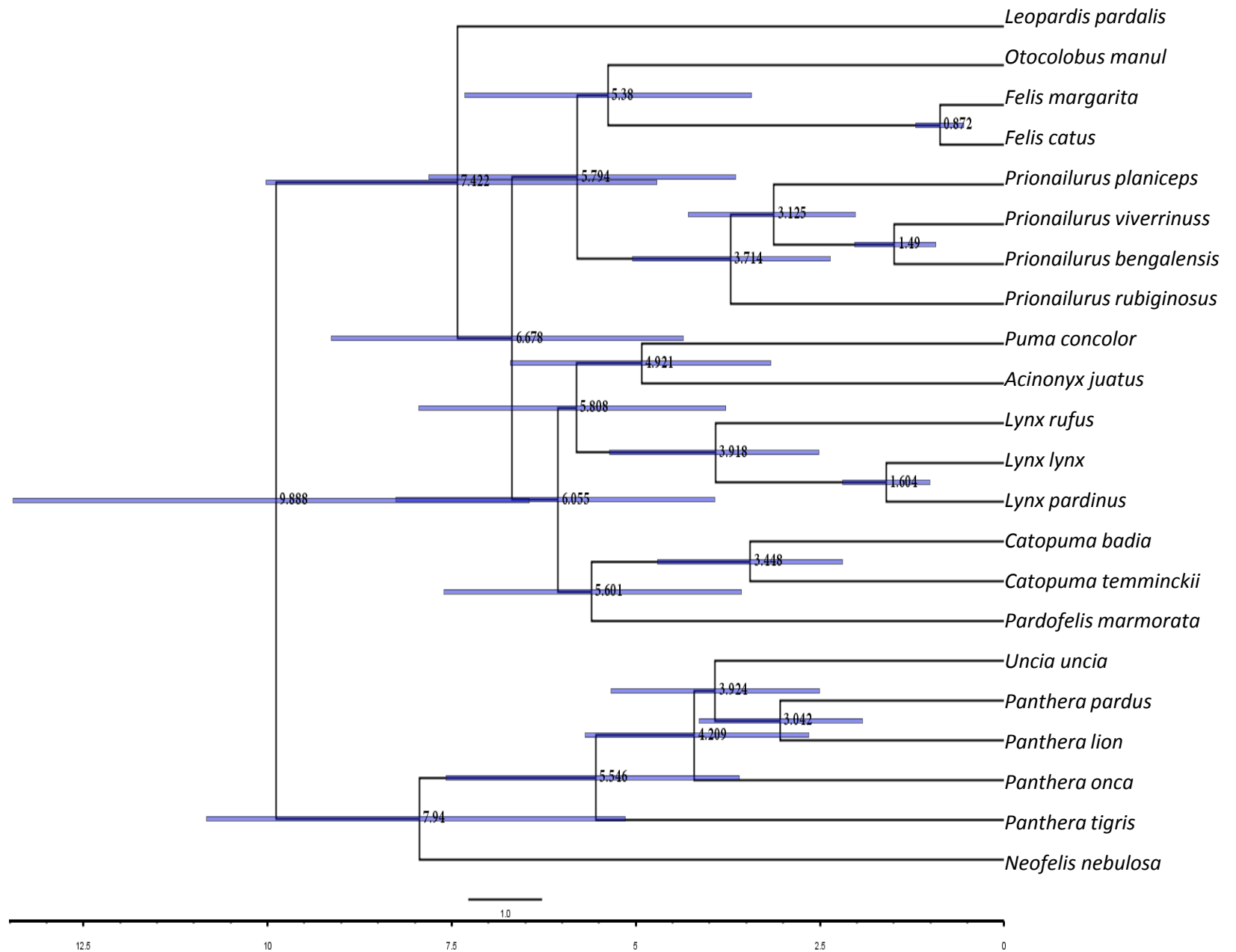

Supplement: Figure S3: Phylogenetic tree with molecular dating [file rsos160350supp4.pdf]
